# Supplementary material for: Multi‐dataset identification of innovative feature genes and molecular mechanisms in keratoconus
Source: J Cell Mol Med. 2024 Sep 19;28(18):e70079. doi: 10.1111/jcmm.70079 (PMC11412914; doi:10.1111/jcmm.70079)
Supplement: Supplementary file 2 — Material S2. [file JCMM-28-e70079-s002.zip › 7.GSEA analysis/3.DBNDD1/KEGG_GLYCOSAMINOGLYCAN_BIOSYNTHESIS_HEPARAN_SULFATE.html]

Details for gene set KEGG\_GLYCOSAMINOGLYCAN\_BIOSYNTHESIS\_HEPARAN\_SULFATE[GSEA]

|  || Dataset | DBNDD1.group.cls#h\_versus\_l |
| Phenotype | group.cls#h\_versus\_l |
| Upregulated in class | h |
| GeneSet | KEGG\_GLYCOSAMINOGLYCAN\_BIOSYNTHESIS\_HEPARAN\_SULFATE |
| Enrichment Score (ES) | 0.21195835 |
| Normalized Enrichment Score (NES) | 0.69713134 |
| Nominal p-value | 0.90831554 |
| FDR q-value | 1.0 |
| FWER p-Value | 1.0 |
Table: GSEA Results Summary

  

Fig 1: Enrichment plot: KEGG\_GLYCOSAMINOGLYCAN\_BIOSYNTHESIS\_HEPARAN\_SULFATE      
 Profile of the Running ES Score & Positions of GeneSet Members on the Rank Ordered List

  

| SYMBOL | TITLE | RANK IN GENE LIST | RANK METRIC SCORE | RUNNING ES | CORE ENRICHMENT || 1 | HS3ST3A1 | na | 876 | 0.396 | 0.1509 | Yes |
| 2 | HS6ST3 | na | 5212 | 0.173 | 0.1029 | Yes |
| 3 | NDST1 | na | 5498 | 0.164 | 0.1677 | Yes |
| 4 | HS3ST2 | na | 6530 | 0.136 | 0.1985 | Yes |
| 5 | HS3ST5 | na | 7740 | 0.109 | 0.2120 | Yes |
| 6 | EXTL1 | na | 9227 | 0.082 | 0.2057 | No |
| 7 | HS6ST1 | na | 10806 | 0.060 | 0.1871 | No |
| 8 | XYLT1 | na | 11667 | 0.050 | 0.1847 | No |
| 9 | HS3ST3B1 | na | 14361 | 0.027 | 0.1193 | No |
| 10 | B3GALT6 | na | 14781 | 0.024 | 0.1181 | No |
| 11 | EXTL3 | na | 15979 | 0.016 | 0.0907 | No |
| 12 | HS3ST1 | na | 16067 | 0.015 | 0.0950 | No |
| 13 | B4GALT7 | na | 17058 | 0.009 | 0.0705 | No |
| 14 | NDST2 | na | 19770 | -0.011 | -0.0028 | No |
| 15 | B3GAT1 | na | 21566 | -0.028 | -0.0422 | No |
| 16 | XYLT2 | na | 21761 | -0.030 | -0.0347 | No |
| 17 | B3GAT3 | na | 21930 | -0.031 | -0.0256 | No |
| 18 | EXT2 | na | 23223 | -0.046 | -0.0424 | No |
| 19 | HS6ST2 | na | 23759 | -0.053 | -0.0342 | No |
| 20 | NDST3 | na | 24293 | -0.060 | -0.0228 | No |
| 21 | HS2ST1 | na | 24650 | -0.066 | -0.0039 | No |
| 22 | EXTL2 | na | 24779 | -0.068 | 0.0225 | No |
| 23 | GLCE | na | 26029 | -0.088 | 0.0256 | No |
| 24 | EXT1 | na | 27153 | -0.111 | 0.0426 | No |
| 25 | B3GAT2 | na | 29281 | -0.165 | 0.0549 | No |
| 26 | NDST4 | na | 31227 | -0.230 | 0.1009 | No |
Table: GSEA details [plain text format]

  

Fig 2: KEGG\_GLYCOSAMINOGLYCAN\_BIOSYNTHESIS\_HEPARAN\_SULFATE      
 Blue-Pink O' Gram in the Space of the Analyzed GeneSet

  

Fig 3: KEGG\_GLYCOSAMINOGLYCAN\_BIOSYNTHESIS\_HEPARAN\_SULFATE: Random ES distribution      
 Gene set null distribution of ES for **KEGG\_GLYCOSAMINOGLYCAN\_BIOSYNTHESIS\_HEPARAN\_SULFATE**
